# Leptomeningeal Metastatic L858R *EGFR*-mutant Lung Cancer: Prompt Response to Osimertinib in the Absence of T790M-mutation and Effective Subsequent Pulsed Erlotinib

**DOI:** 10.2147/OTT.S336012

**Published:** 2022-06-14

**Authors:** Aladdin Kanbour, Faroug Salih, Wafa Abualainin, Mohamed Abdelrazek, Lajos Szabados, Issam Al-Bozom, Nabil E Omar

**Affiliations:** 1 Medical Oncology Department, National Center for Cancer Care and Research, Hamad Medical Corporation, Doha, Qatar; 2 Solid Tumor Section, Molecular Genetics Laboratory, Diagnostic Genomic Division, Department of Laboratory Medicine and Pathology, Hamad Medical Corporation, Doha, Qatar; 3 Clinical Imaging Department, National Center for Cancer Care and Research, Hamad Medical Corporation, Doha, Qatar; 4 Precision Medicine Section, Anatomical Pathology Department, Department of Laboratory Medicine and Pathology, Hamad Medical Corporation, Doha, Qatar; 5 Pharmacy Department, National Center for Cancer Care and Research, Hamad Medical Corporation, Doha, Qatar

**Keywords:** NSCLC, non-small-cell lung cancer, LMC, leptomeningeal carcinomatosis, *EGFR*, epidermal growth factor receptor, *EGFR*-TKIs, *EGFR* tyrosine kinase inhibitors, osimertinib, pulsed erlotinib

## Abstract

Leptomeningeal carcinomatosis (LMC) is a known sequel of metastatic lung cancer and its treatment is challenging. Nevertheless, treatment options for LMC due to metastatic epidermal growth factor receptor-mutant (*EGFR*-mutant) lung adenocarcinoma are expanding. We present a 52-year-old male patient with metastatic non-small-cell lung cancer (NSCLC). The patient was found to have L858R mutation in exon 21 of the EGFR gene. He was initially treated with erlotinib, followed by afatinib/cetuximab, followed by chemotherapy. Thereafter, his disease progressed to LMC. Although tissue biopsy did not show T790M-mutation, osimertinib (160 mg once daily) promptly induced clinical and radiological response that continued for five months. High dose pulsed erlotinib (1500 mg weekly) improved his quality of life and extended his survival for a further four months.

## Video abstract

**Figure:**
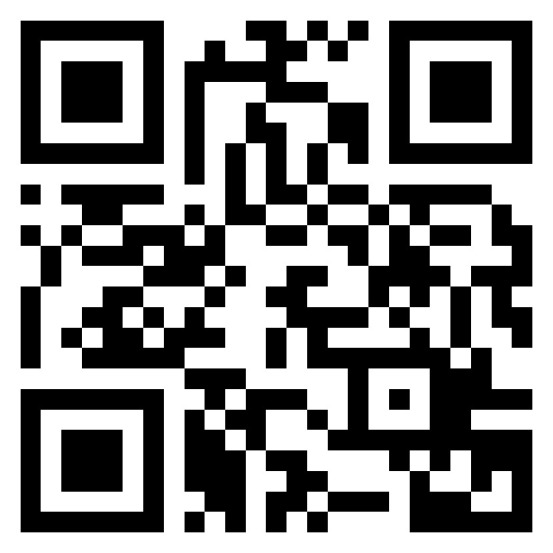

Point your SmartPhone at the code above. If you have a QR code reader the video abstract will appear. Or use:

https://youtu.be/hB2KtcuyWz4

## Introduction

The incidence of LMC in NSCLC has increased due to improved survival of patients from advances in systemic therapy;^1^ LMC is estimated to be present in about nine percent of EGFR-mutant NSCLC patients.^2^,^3^ The most common mutations in NSCLC patients that have been identified in the *EGFR* gene are in-frame deletion in exon 19 and L858R in exon 21. L858R mutation is a single nucleotide variant in exon 21 in the tyrosine kinase domain of the *EGFR* gene. This missense mutation located at the coding position 2573, causing nucleotide change from thymine (T) to guanine (G) causing amino acid substitution from leucine (L) to arginine (R) at codon 858. *EGFR* mutations are more frequent in adenocarcinoma than other NSCLCs, in females, and in never smokers.^4^,^5^

As demonstrated in previous studies, *EGFR* tyrosine kinase inhibitors (*EGFR*-TKIs) can improve prognosis of these patients.^6^,^7^ However, most lung adenocarcinoma patients with *EGFR*-mutations who are treated with *EGFR* tyrosine kinase inhibitors will relapse because of resistance development against the used drug.^8^ The most common resistance mechanism is acquisition of the *EGFR* exon 20 T790M mutation.

Tumors that develop resistance to *EGFR*-TKIs, mostly due to *EGFR*-T790M mutation, represent a challenge for durable response.^9^

Osimertinib, a third-generation *EGFR*-TKI, is an approved agent for previously treated *EGFR*-TKIs mutant NSCLC patients who develop *EGFR*-T790M resistance mutation,^10–13^ and untreated *EGFR*-mutant advanced NSCLC patients.^14^

The updated results from BLOOM study indicate that osimertinib is effective for LMC, regardless of T790M-mutation status.^15^,^16^ Furthermore, intermittent high, rather than standard, dose of erlotinib reaches therapeutic concentrations within the cerebrospinal fluid (CSF) and is well tolerated in patients with LMC.^17^ Herein, we present a patient with T790M-negative metastatic NSCLC adenocarcinoma who developed LMC. The patient’s symptoms responded promptly well to osimertinib followed by pulsed erlotinib and showed radiological improvement that lasted for a total of nine months.

## Case Report

A 52-year-old, nonsmoker, Asian male was first diagnosed with lung cancer in February 2017. His initial presentation was persistent coughing. Imaging showed a 4 cm sized left lung lesion lateral to the left lung hilum. Staging workup showed mediastinal (N2) lymph node, left adrenal involvement and multiple bone metastases with minimal left pleural fluid. A tissue biopsy of his lung mass revealed NSCLC adenocarcinoma; *EGFR*-mutant with L858R in exon 21, PD-L1 negative and ALK rearrangement negative.

**Figure 1:**
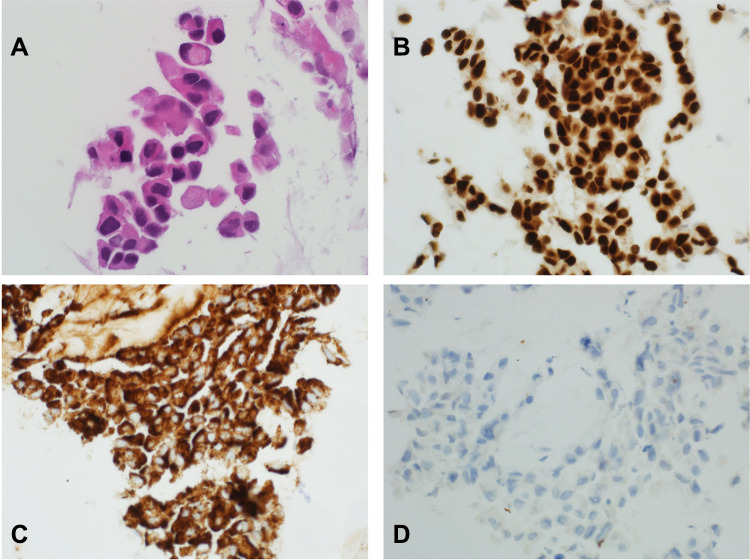
(**A**) Microscopic appearance of the tumor characterized by solid sheets of highly atypical cells (H&E ×600). (**B**) Immunohistochemistry stain against TTF-1 shows strong nuclear positivity (immunohistochemistry ×400). (**C**) Immunohistochemistry stain against napsin-A shows strong cytoplasmic positivity (immunohistochemistry ×400). (**D**) Immunohistochemistry stain against PD- L1 shows a negative staining (immunohistochemistry ×400).

Pathological sections showed fragments of skeletal muscle fibers and fibroadipose tissue infiltrated by a tumor characterized by ill-defined glandular structures as well as showing solid sheets of pleomorphic polygonal cells some with cytoplasmic mucin (Figure 1A). These tumor cells are strongly positive for CK 7, TTF-1 (Figure 1B) and napsin-A (Figure 1C) while negative with CK 20, CD X2, PAPH and PSA. PD-L1 (TPS) is negative (Figure 1D). ALK 5 A4 by immunohistochemistry is negative.

The *EGFR* mutation was confirmed by molecular technique. The tumor area was marked by consultant pathologist on a formalin-fixed paraffin-embedded (FFPE) slide. Genomic DNA was extracted and analyzed by using *EGFR*-RT52 kit (EntroGen, USA). The kit is based on a real time-PCR qualitative genotyping assay that uses fluorescently labeled probes for detection of 30 recurrent mutations G719X in exons 18, deletions in exon 19, T790M, S768I and insertions in exon 20 (resistance mutations) and L858R and L861Q in exon 21 of *EGFR* gene. This test will not identify rare mutations, such as E709K and D770-N771ins.

The patient initially received erlotinib 150 mg then reduced to 100 mg because intolerance in the form of acne and mucositis; he had clinical and metabolic response until May 2018: a progression-free survival of 18 months.

In August 2018, positron emission tomography (PET) scan showed disease progression in the bones with stationary lung lesions. Liquid biopsy was tested and did not demonstrate T790M-mutation. At that point of his treatment, afatinib and cetuximab were introduced but then discontinued two weeks later, as he developed grade 3–4 oral/nasal mucositis. Carboplatin and pemetrexed were given as third line, and CT scan showed stationary course of the disease for another 12 months.

**Figure 2:**
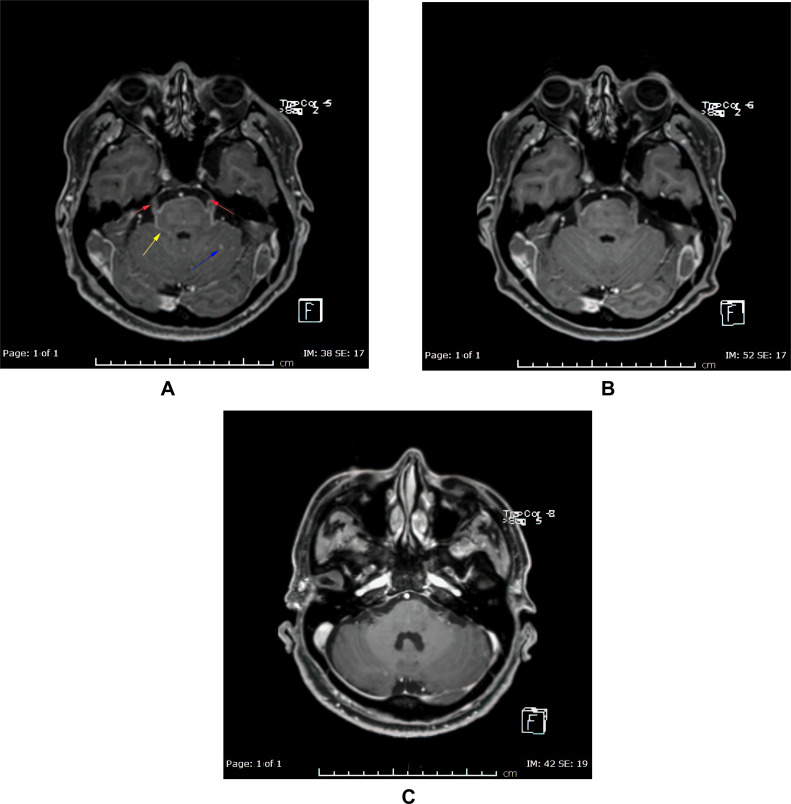
Axial post contrast fat saturated images of the brain: before (**A**) and 14 days (**B**), and three months (**C**) post-osimertinib. (**A**) Before treatment showing multiple small enhancing foci in the left cerebellar hemisphere (blue arrow), abnormal enhancement involving the perimesencephalic cistern (yellow arrow) and abnormal enhancement of the trigeminal nerve bilaterally (red arrows). (**B**) After treatment showing almost complete clearance of the abovementioned nodules in left cerebellar hemisphere, the abnormal enhancement in the perimesencephalic cistern and the trigeminal nerves. (**C**) After three months treatment showing persistent improvement with no detected nodules or meningeal enhancement.

**Figure 3:**
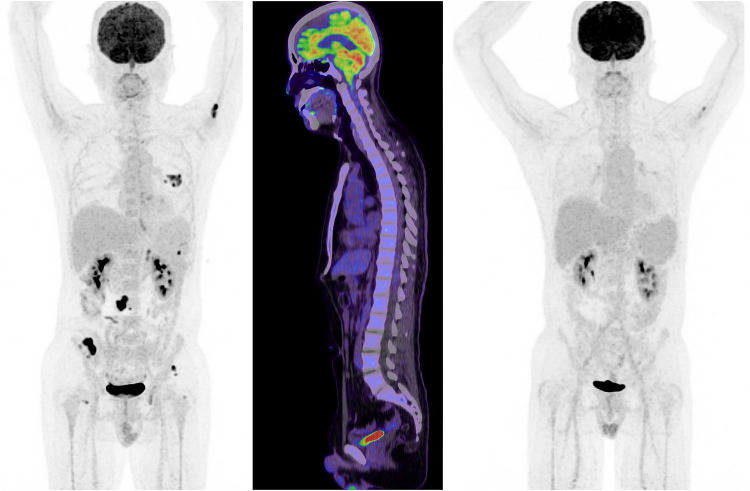
18F-FDG PET/CT maximum intensity projection (MIP) images and sagittal fused PET/CT images. Staging 18F-FDG PET/CT maximum intensity projection (MIP) image (left side) showing FDG-avid left lung, adrenal lesion and some bone metastases (left humerus, right iliac wing). Sagittal fused PET/CT image (middle image) showing no evidence of meningeal involvement. Follow-up PET/CT MIP image (right side) demonstrates near complete resolution of lung, adrenal and right iliac wing lesion as well as decreasing uptake in the left humerus.

**Figure 4:**
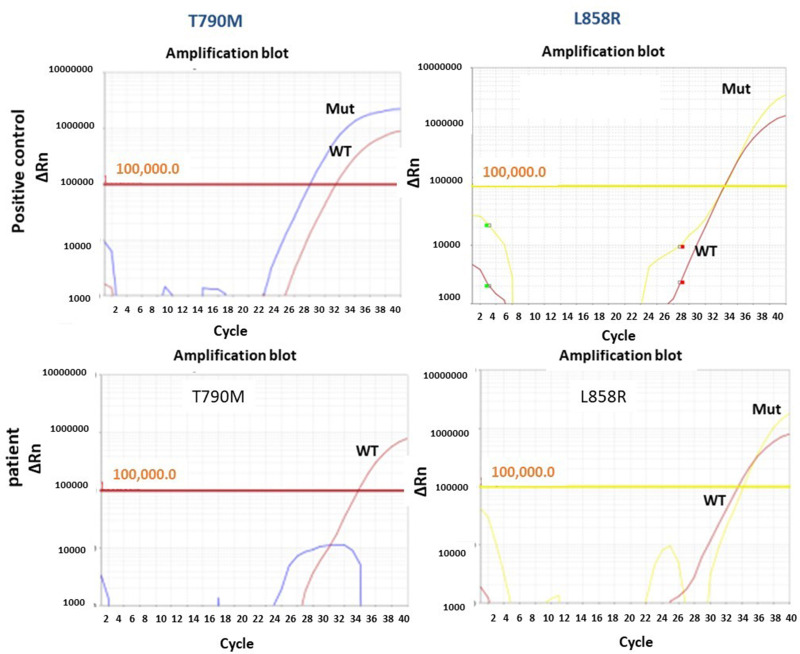
Real time-PCR analysis for EGFR hotspots, real time-PCR results of *EGFR* gene for L858R and T790M. The DNA was extracted from FFPE using Promega kit following manufacturer instructions (Cat No: AS1130) and the results obtained using Entrogen kit for *EGFR* mutation analysis (*EGFR*-RT52) and analyzed by Quant Studio Dx in accordance with CAP guidelines for molecular genetics laboratory.

In October 2019, the patient presented to the Emergency Department complaining of new onset severe generalized headache, dizziness, blurred vision, and repeated vomiting evolving over two weeks. Examination revealed neck rigidity without papilledema, intact cranial nerves, power 5/5 in both upper and lower limbs, reflexes +1, and downgoing plantar response. Magnetic resonance imaging (MRI) of the brain showed abnormal sulcal and cisternal enhancement representing extensive leptomeningeal metastases, with small cerebellar and cerebral cortical metastases (Figure 2A). Whole body PET scan showed disease progression (Figure 3). Patient refused a lumbar puncture. He received dexamethasone 8 mg twice daily for three days along with paracetamol and ondansetron without symptom improvement. The patient was given osimertinib as a replacement of his treatment option, 160 mg once daily, osimertinib was started on day four, all his symptoms have improved dramatically within the following 24 h. On day six, a tissue biopsy of a right pelvic bone lesion showed metastatic adenocarcinoma of the lung. The T790M-mutation was not detected in this biopsy (Figure 4). Fourteen days post osimertinib initiation, MRI of the brain showed complete clearance of some tumor lesions and decrease in size of other lesions in the brain. Furthermore, a decrease in the leptomeningeal regions (Figure 2B). A month later, November 2019, a follow-up PET scan showed favorable response (Figure 3), and in January 2020 a follow-up MRI for brain and spine showed regression of the tiny cerebral enhancing metastatic lesions (Figure 2C).

In February 2020, headache and vomiting recurred but generally the patient complained of less severe symptoms than the symptoms presented in October 2019. CSF examination showed atypical cells highly suspicious for metastatic malignancy. MRI spine showed diffuse meningeal thickening, and subtle nodularity along the lumbar spinal cord and thecal sac representing metastases.

PET scan showed practically stable presentation (Figure 3). Osimertinib was stopped at this point. The patient was followed with a trial of intrathecal methotrexate, twice weekly for two weeks, which induced severe post lumbar puncture headache without relieving his symptoms. Palliative whole brain radiotherapy and meningeal radiation were discussed with the patient several times as an additional treatment option, but he refused.

**Figure 5:**
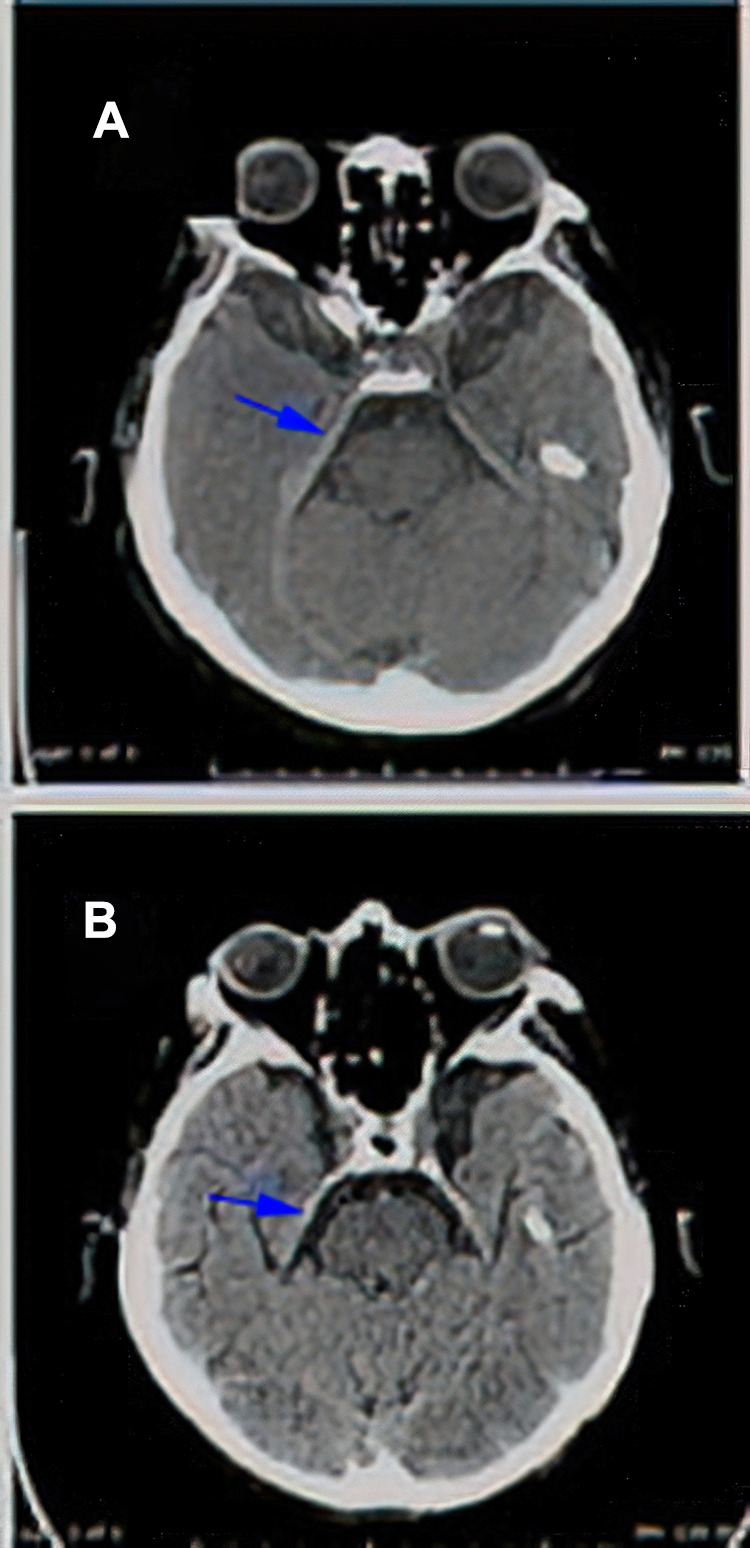
(**A**) Axial CT scan of the brain without IV contrast (patient cannot do MRI and cannot receive IV contrast because of rising creatine), this limited study shows thickening of the right tentorium in comparison to left one suggesting meningeal involvement (arrow in **A**). (**B**) Axial CT scan of the brain without IV contrast (patient cannot do MRI and cannot receive IV contrast because of rising creatinine), shows almost complete clearance of the thickened right tentorial leaflet (arrow in **B**).

By the end of March 2020, the patient was given pulsed erlotinib 1050 mg once weekly increased to 1500 mg one week later. In April, the patient showed up following a syncope and fall, CT scanning was performed for the head and showed changes related to the leptomeningeal metastasis with thickening of the tentorium cerebelli and along the right greater wing of sphenoid (Figure 5A). In July, while presenting with seizures, CT scanning of the head showed reduction in the leptomeningeal and tentorium cerebelli thickening (Figure 5B). He continued pulsed erlotinib for almost four months, as his overall symptoms had improved without significant medication side effect. Unfortunately, his performance status kept deteriorating and, unfortunately; he died by the end of July 2020. Overall, he survived almost nine months post LMC diagnosis: five months on osimertinib and four months on pulsed erlotinib.

## Discussion

Metastatic NSCLC and LMC have been reported with poor prognosis.^1^ Our patient had stage IV NSCLC adenocarcinoma with *EGFR*-mutation L858R in exon-21. His first line was conventional dose erlotinib with good partial metabolic response for 18 months. Following disease progression second line was tried as afatinib/cetuximab but discontinued after two weeks only due to poor tolerance and grade 3–4 oral/nasal mucositis. Furthermore, third-line chemotherapy with carboplatin/pemetrexed was used and was effective for further 12 months. Unfortunately, he developed LMC and new systemic lesions were detected without showing T790M-mutation from a metastatic bone site. Since his tumor carried an *EGFR*-mutation, the decision to start osimertinib was made, to overcome the relapse and progression as several studies confirmed that the osimertinib has possible effective outcome even if the tumor was T790M-negative.

First generation TKIs erlotinib and gefitinib have shown some response in the treatment of LMC in small studies and case series.^18–21^ Osimertinib, a third-generation TKI, is superior over standard platinum-based chemotherapy for the treatment of patients with T790M-positive disease who had progressed after first-line *EGFR*-TKI.^12^,^13^

It is also superior over first generation TKIs in untreated patients regardless of T790M status.^14^ The update from the BLOOM study which included 21 unselected patients and 11 T790M-positive patients concluded that osimertinib penetrates the blood–brain barrier.^16^ Ballard et al added that osimertinib penetration to the blood–brain barrier is better than gefitinib, afatinib or rociletinib.^22^ Osimertinib presents encouraging activity and manageable tolerability in patients with leptomeningeal metastases from *EGFR*-mutant NSCLC observed at 160 mg a day.^16^ The findings from BLOOM study was observed in several case reports (Table 1).^15^,^23–25^

**Table 1:** Summary of Previous Literature of Osimertinib 160 mg for Treatment of Patients with Leptomeningeal Metastases from *EGFR*-Mutant NSCLC

Ref. Number	Number of Patients	Gender (M/F)	Ethnicity	Age	T709M Status	Treatment Used for LMC Before High-dose Osimertinib (160 mg)	Median DoR to Osimertinib 160 mg (Months)
[12]	41	12 M 29 F	Asian	59 (44–75)	Unselected (n=21) T790M-positive (n=20)	Prior intrathecal chemotherapy (n=18)	8.3 (5.6 to 16.5)
[20]	1	F	Asian	59	Negative	Osimertinib 80 mg	3
[21]	1	M	Asian	56	Negative	WBRT Osimertinib 80 mg	6
[22]	3 A, B, C	F	Asian	51 60 56	Positive Positive Negative	None Osimertinib 80 mg Osimertinib 80 mg	8 7 12
Current case	1	M	Asian	52	Negative	None	5

**Abbreviations**: LMC, leptomeningeal carcinomatosis; DoR, duration of response.

Our patient demonstrated a prompt dramatic clinical response (within 24 h of commencing osimertinib) with significant response shown in brain imaging. Even though the T790M was negative based on bone biopsy, which might be a false negative,^26^,^27^ but in our case the chance of having a false negative was low as the bone biopsy showed acceptable DNA quality. In addition, the L858R mutation was detected at this metastatic site. The prompt, relatively sustained improvement in the patient condition following treatment with osimertinib should not be ignored. Our case report has shown great benefit of the sequential treatment strategy that we applied to manage our patient, including erlotinib (18 months), afatinib/cetuximab, chemotherapy with carboplatin/pemetrexed (12 months), osimertinib radiological response that continued for (5 months) and finally high-dose pulsed erlotinib (additional 4 months). Osimertinib has shown dramatic quick effective and well-tolerated therapeutic option for LMC in *EGFR*-mutant NSCLC, even in T790M-negative disease, our case showed response within 24 h post-osimertinib initiation.

The overall treatment time since the patient was first diagnosed is approximately four years. Moreover, he responded to pulsed erlotinib for additional four months. Median survival of *EGFR*-mutated NSCLC patients with LMC was 3.1 months in previous studies,^2^,^3^ while our patient had survived almost nine months post LMC diagnosis as he was under selective sequential treatment.

We have managed this patient with different treatment options first to reduce the disease progression according to the molecular results and second to overcome any developed resistance, taking into consideration the patient status and performance. Patients with LMC are known to have poor performance status; a treatment that is tolerable with fewer side effects should always be taken into consideration.

## Conclusion

Osimertinib can result in clinical and radiological response, with extended survival when used for *EGFR*-mutant T790M-negative lung adenocarcinoma progressed to LMC. Pulsed erlotinib can be one of the options to help in controlling the symptoms of LMC and extension of the patient’s survival.

## Data Sharing Statement

The datasets used and/or analyzed during the current study are available from the corresponding author on reasonable request.
